# From Strength to Dexterity: Clinically Meaningful Recovery of Upper Limb in Individuals with Cervical Spinal Cord Injury

**DOI:** 10.3390/jcm15072633

**Published:** 2026-03-30

**Authors:** Federica Tamburella, Diego Piatti, Stefano Filippo Castiglia, Claudia Celletti, Giada Serratore, Giorgio Scivoletto

**Affiliations:** 1 Department of Life Sciences, Health and Health Professions, Link Campus University, 00165 Rome, Italy; f.tamburella@unilink.it (F.T.); stefanofilippo.castiglia@uniroma1.it (S.F.C.); c.celletti@unilink.it (C.C.); 2 Laboratory of Spinal Neurorehabilitation, IRCCS Santa Lucia Foundation, 00179 Rome, Italy; g.serratore@hsantalucia.it; 3 Laboratory of Neuromotor Physiology, IRCCS Santa Lucia Foundation, 00179 Rome, Italy; 4 Department of Medico-Surgical Sciences and Biotechnologies, “Sapienza” University of Rome-Polo Pontino, 04100 Latina, Italy; 5 Ospedale CTO Andrea Alesini, ASL ROMA 2, 00145 Rome, Italy; giorgio.scivoletto@aslroma2.it

**Keywords:** spinal cord injuries, tetraplegia, upper limb, functional recovery, neurological assessment, activities of daily living

## Abstract

**Background**: Understanding the temporal relationship between neurological and functional recovery after cervical spinal cord injury (SCI) is crucial for optimizing rehabilitation timing and clinical interpretation. This prospective longitudinal study aimed to investigate the temporal dynamics of clinically meaningful neurological and functional recovery in individuals with subacute SCI during inpatient rehabilitation. **Methods:** We enrolled 21 individuals with incomplete cSCI (AIS C and D). Evaluations were performed every 15 days, from admission up to 120 days. Recovery was defined using the Time to First Improvement based on thresholds exceeding the Minimal Detectable Change or Minimal Important Difference) for neurological scales (Upper Extremities Motor Score—UEMS, Graded Redefined Assessment of Strength, Sensation and Prehension—GRASSP subtests for Strength and Sensation) and the Minimal Clinically Important Difference for functional scales (Spinal Cord Independence Measure, GRASSP Ability and Prehension Performance). Survival analysis (Kaplan–Meier) and pairwise comparisons were used to analyze the temporal sequence of recovery. **Results**: Neurological and functional recovery showed a parallel macro-evolution. However, granular analysis revealed that motor strength improved significantly earlier than sensory recovery and fine motor dexterity. No significant differences were found between dominant and non-dominant limbs. **Conclusions**: Upper limb recovery follows a phase-specific evolution where motor strength provides the substrate for functional gains supporting a phase-specific approach to rehabilitation.

## 1. Introduction

Spinal cord injury (SCI) represent a complex condition that has a significant impact on the individual’s life, making the rehabilitation process crucial for the recovery of impaired functions [1]. The care of individuals with SCI requires a multidisciplinary approach that addresses the heterogeneity of clinical presentations, multisystem involvement, and secondary health conditions [2]. Neurorehabilitation is a core component of post-SCI care [3], representing a holistic and multidisciplinary process that aims to compensate for functional impairments, optimize preserved sensorimotor functions, and address recovery within damaged neural circuits, ultimately promoting the highest possible level of autonomy and facilitating full social and occupational reintegration [4].

Data from the Global Burden of Disease 2021 study estimate that approximately 14.5 million people worldwide are living with SCI, including 7.30 million individuals with cervical injuries [5]. Given the high prevalence of cervical SCI, rehabilitation represents a fundamental component of care. In this population, upper limb involvement is frequently characterized by reduced sensorimotor abilities that significantly affect the performance of daily living activities requiring arm and hand function [6]; accordingly, rehabilitation may play a crucial role in upper limb functional recovery [7], as regaining arm and hand function is considered a priority for individuals with cervical lesions [8].

Neuroplasticity, defined as the ability of the nervous system to reorganize by forming new neural connections [9], plays a critical role in promoting structural and functional recovery and enables potential functional compensation after injury. Such compensation enables patients to achieve functional independence by adapting to the loss of voluntary muscle control below the level of the lesion and utilizing alternative strategies or assistive devices, also considering the new opportunities offered by 3D-printing technologies [10]. This is particularly significant in severe SCI cases, where voluntary motor function is absent below the level of injury [11]. Therefore, reorganization of anatomical structures and functional circuits both above and below the injury site underlies the potential for differential levels of functional recovery [12].

Curt et al. defined neurological recovery as the result of reorganization within injured neural circuits and of repair mechanisms such as remyelination, regeneration, or reconnection of fibers damaged by SCI. Functional recovery, on the other hand, is defined as the result of compensatory mechanisms and the development of strategies adopted by the individual through neuromotor rehabilitation, reflecting changes in the management of activities of daily living (ADLs) [13]. Authors also reported that individuals with complete SCI showed improvements in ADLs (i.e., functional recovery) largely independent of neurological changes (i.e., without changes in upper limb motor scores), whereas those with incomplete SCI exhibited greater functional and neurological recovery. This recovery was not associated with improved spinal conductivity, supporting the assumption that functional gains primarily rely on compensatory mechanisms in complete SCI and on neural plasticity in incomplete SCI [13].

Neurological examination of individuals with SCI is typically performed according to the International Standards for Neurological Classification of Spinal Cord Injury (ISNCSCI) [14], which is considered the gold standard for determining neurological level of injury and severity of impairment. One component of the ISNCSCI is the Upper Extremity Motor Score (UEMS), which is frequently used in clinical research to investigate the course of neurological recovery. However, the UEMS is limited to the assessment of only five key muscle groups for each upper limb. Consequently, this motor score may be insufficient for prognostic purposes and for detecting therapeutic benefits, whether rehabilitative or pharmacological, as it is affected by both floor and ceiling effects [15].

To provide more sensitive and comprehensive assessments of upper limb recovery in cervical SCI, the Graded Redefined Assessment of Strength, Sensation and Prehension (GRASSP) has been developed [16]. GRASSP is recommended for use in the early acute phases to any point in the post-injury time-course, particularly when a change in neurological status is the construct of interest. The GRASSP improves clinical, neurological, and functional evaluation of the upper limb by incorporating multiple subtests aimed at assessing sensation, complex and fine hand movements, and muscle strength across a broader range of upper limb muscles [17]. Moreover, the GRASSP demonstrates excellent psychometric properties, enabling accurate characterization of recovery profiles over time [18].

While the UEMS represents a purely neurological assessment and the GRASSP captures both neurological and functional aspects, the Spinal Cord Independence Measure II (SCIM) [19] is specifically designed to evaluate functional recovery, reflecting the impact of motor recovery on upper limb functionality in terms of ADLs management and self-care. The SCIM is composed of three primary domains (self-care, respiration and sphincter management, and mobility), further subdivided into 18 distinct subitems. Both domains and subitems are weighted based on their clinical relevance to overall functional performance in the SCI population [20]. Importantly, the contribution of each subscale differs depending on the clinical objective. In individuals with cervical SCI, improvements in the total SCIM score are particularly meaningful when driven by gains in the Self-care subscale, which includes activities such as opening containers, cutting food, dressing, and personal hygiene, all closely related to upper limb function.

To date, few studies in individuals with cervical SCI have investigated the longitudinal evolution of neurological and functional recovery with evaluations performed at relatively long time intervals (e.g., every six months) [21], potentially overlooking critical phases of recovery. Furthermore, existing literature rarely considers neurorehabilitation as a variable of interest, reporting recovery trajectories without systematically accounting for the type, intensity, or content of rehabilitation interventions provided. As a result, the temporal relationship between neurological and functional recovery, as well as the contribution of conventional physiotherapy and occupational therapy, remains poorly understood.

In light of this, the aim of this prospective longitudinal open-cohort study was to investigate the temporal dynamics of clinically meaningful neurological and functional recovery in individuals with tetraplegia during inpatient rehabilitation in an Italian Spinal Cord Unit. Specifically, we examined whether improvement in neurological measures tended to precede, overlap with, or follow improvement in functional measures. The study was not designed to test the effect of specific rehabilitation interventions, but rather to characterize the timing and sequencing of recovery under standard treatment conditions. The primary objective was to establish whether neurological recovery precedes functional recovery or vice versa. This distinction is clinically relevant, as functional recovery—being largely driven by compensatory mechanisms and strategy adoption—may emerge independently of, or even prior to, neurological recovery, which is more directly related to neural plasticity and repair processes. The study also assessed whether, at discharge, functional recovery exceeds neurological recovery or follows a comparable trajectory. Understanding the temporal interplay between neurological and functional recovery is expected to provide clinically meaningful information to guide rehabilitation timing, goal-setting, and prioritization of therapeutic interventions during inpatient rehabilitation.

## 2. Materials and Methods

This prospective longitudinal open-cohort study was conducted at the I.R.C.C.S. Fondazione Santa Lucia (FSL) of Rome (Italy) for Research and Healthcare and received formal approval from the Local Independent Ethics Committee (protocol no. CE/PROG.779). All study procedures were performed in accordance with national and institutional guidelines for human research, as well as the World Medical Association’s Declaration of Helsinki. All participants provided written informed consent for the publication of data derived from their participation.

### 2.1. Individuals with SCI Cohort

All individuals admitted to the Spinal Cord Unit of FSL were screened for eligibility. Demographic and clinical characteristics were collected at admission in the Spinal Cord Unit and were recorded by an experienced neurologist. In detail following data were collected: age, gender, SCI etiology, time since injury, American Spinal Injury Association Impairment Scale (AIS) neurological level, and functional capabilities in the ADLs based on the SCIM. Participants with cervical SCI classified as AIS grade C or D were included in the study. Exclusion criteria included respiratory dependence and the presence of severe non-spinal neurological damage associated with upper limb impairment (e.g., brachial plexus injury, severe traumatic brain injury, …).

### 2.2. Clinical Assessment

From hospital admission until discharge, participants underwent neurological and functional assessments of both upper limbs every two weeks assessed by a specialized neurologist together with a physiotherapist. To minimize inter-clinician variability, all assessments were performed by same clinicians at each time point. The individual’s dominant upper limb was defined per the Edinburgh Handedness Inventory-Short Form [22].

The neurological recovery was assessed per the:

- Upper Extremity Motor Score (UEMS), a component of the International Standards for Neurological Classification of SCI (ISNCSCI) [23], that specifically evaluates voluntary motor function of the upper limbs. The UEMS assesses the strength of five key muscle groups per side—corresponding to the C5–T1 myotomes—using a standardized 6-point ordinal scale (0–5). The total UEMS ranges from 0 to 50, with higher values indicating better motor preservation. UEMS provides a quantitative measure of motor impairment and neurological recovery in the upper limbs, however it primarily reflects isolated muscle strength and does not capture hand function, dexterity, or the ability to perform functional tasks in daily activities.
- Strength and Sensation subtests of GRASSP [18,24] scale performed separately for each hand. The GRASSP is composed of four subtests designed to provide a comprehensive evaluation of upper limb impairment in individuals with cervical SCI. Strength subtest assesses voluntary muscle force of key upper limb muscles using a 0–5 ordinal scale, yielding a maximum score of 50 for each upper limb, and Sensation subtest evaluates palmar Sensation through the assessment of soft touch, with scores reflecting the integrity of sensory input relevant to hand function into three specific zones. Total Sensation score ranges from 0 to 24, with higher scores indicating better sensory integrity relevant to hand function.

The functional recovery was analyzed according to:

- Grasp subtests of GRASSP scale performed separately for each hand to assess (1) Prehension Ability (GRASSP-PA), examining the quality and coordination of three grasp patterns, focusing on the appropriateness of hand configuration, finger movement, and thumb–finger interaction rather than speed. Each grasp pattern (Cylindrical grasp, Tip to tip pinch and Lateral key pinch) is scored using a 0–4 ordinal scale, resulting in a Qualitative Prehension score ranging from 0 to 12 per hand, with higher scores indicating better quality of prehension and grasp coordination; (2) Prehension Performance (GRASSP-PP), measuring task performance through timed object manipulation tasks focusing on cylindrical grasp, tripod grasp, tip-to-tip pinch, and lateral key pinch. Each task is scored based on the time required to successfully complete the task, using a 0–5 ordinal scale, where higher scores indicate faster and more efficient performance.
- SCIM [19] is a disease-specific scale designed to assess functional independence in individuals with SCI during ADLs. It evaluates three domains (self-care, respiration and sphincter management, and mobility) with a total score ranging from 0 to 100, where higher scores indicate greater functional independence.

**Figure 1:**
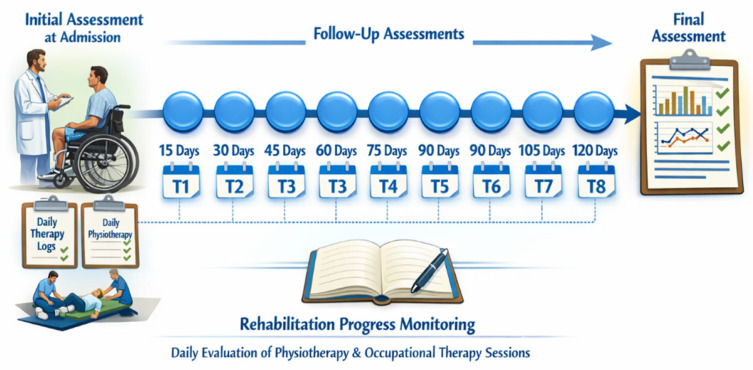
Timeline of clinical assessments during hospitalization. Generative AI was used to refine this figure.

Clinical assessments were conducted at specific time points throughout the rehabilitation process. An initial assessment was performed after individuals with SCI signed the informed consent, followed by repeated evaluations at different subsequent time points. The assessments were conducted according to a predetermined schedule, with the first measurement (T1) taking place approximately 15 days after admission into the Spinal Cord Unit. Subsequent evaluations were carried out every 15 days, specifically at 30 days (T2), 45 days (T3), 60 days (T4), 75 days (T5), 90 days (T6), 105 days (T7), and lastly at 120 days (T2) after admission, in accordance with the hospitalization duration and any problems that arose during hospitalization. Throughout the entire observation period, daily documentation of physiotherapy and occupational therapy sessions delivered was maintained to continuously monitor rehabilitation and provide context for the clinical assessment results (Figure 1).

### 2.3. Intervention

Rehabilitative approach consisted of a multimodal approach including physiotherapy and occupational therapy delivered by experienced physiotherapists working in the Spinal Cord Unit, based on medical prescription. The rehabilitation program was supervised by expert neurologist and physiatrist specialized in SCI rehabilitation. Rehabilitation sessions, both conventional and occupational, started as soon as possible and continued until the end of hospitalization. Treatment prescriptions were individual-specific, and each individual with SCI performed at least two daily individual rehabilitation sessions lasting 40 min each one. While we reported the average number of sessions and the daily frequency of treatment, the ‘treatment-as-usual’ nature of the intervention precludes a formal assessment of the dose–response relationship between specific activities and recovery outcomes. The treatments were carried out according to the standard procedures at the Spinal Cord Injuries unit and as previously studied. Regarding the non-robotic component, they were performed following a prior study conducted at the same hospital [25]. Periodic team meetings with medical doctors and physiotherapists were held at least every 15 days to assess and tailor ad hoc rehabilitation program.

### 2.4. Statistical Analysis

#### 2.4.1. Definition of Domains and Outcome Measures

Recovery was analyzed using a time-to-event framework based on the time to first clinically meaningful improvement (TTFI). Improvement was defined as the first time point at which an individual with SCI showed an increase from baseline (T0) that met or exceeded a predefined threshold. To ensure that these changes reflected a real clinical evolution rather than measurement noise, we adopted thresholds based on the Minimal Detectable Change (MDC), representing the smallest change that can be detected beyond the measurement error, or the Minimal Important Difference (MID), which reflects a clinically meaningful change. Specifically, for the SCIM, we used a threshold of ≥4 points, corresponding to the validated Minimal Clinically Important Difference (MCID) [26]. For the UEMS, a threshold of ≥3 points was set, based on the MDC, to ensure the observed recovery exceeded the intrinsic variability of the scale [27]. For the GRASSP subscales (Strength, Sensation, Prehension Ability, and Performance), we adopted thresholds ≥5 for Strength, ≥4 for Sensation and Prehension) reflecting the MID reported in literature for subacute cervical spinal cord injury, as these values represent a significant clinical progress in upper limb functionality [17]. Despite these technical distinctions between MDC, MID, and MCID, the term MCID is used throughout the remainder of the text for brevity to refer to the achievement of these predefined clinical improvement thresholds [26].

For each individual scale, the baseline was defined as the first available assessment for each participant and limb side, as applicable. Change scores were calculated relative to the baseline. Because the primary objective was to compare the temporal ordering of clinically meaningful improvement within individuals, all analyses were anchored to each participant’s own baseline assessment. This within-patient time-to-improvement framework reduces dependence on absolute baseline scores and allows for the pooling of clinically heterogeneous etiologies to investigate temporal recovery relationships. For limb-specific measures (UEMS and GRASSP subscales), patient-level outcomes were calculated by first computing TTFI separately for each limb and then collapsing to the participant level by keeping the earliest occurrence of MCID-defined improvement across sides. Participants who did not meet the MCID threshold for a given scale were censored at the last available observation. At the domain level, composite TTFI was built by identifying the earliest time point for each participant when any constituent scale within the domain reached its MCID threshold. Participants who did not achieve MCID on any scale within the domain were censored at the most recent observed time across all constituent scales. Given the limited sample size, inference did not rely on Cox regression alone. Time-to-event patterns were first described non-parametrically using Kaplan–Meier curves and restricted mean survival time (RMST) and were complemented by paired within-subject non-parametric analyses (sign test or Wilcoxon signed-rank test when applicable). Cox proportional hazards models were used as secondary inferential summaries with participant-level robust variance estimation and were fitted only when the number of observed events allowed stable estimation. Although the sample size was modest, Cox proportional hazards models were used because they provide a semi-parametric framework that does not require specification of the baseline hazard and remain appropriate for small samples when interpreted cautiously. To improve robustness and account for within-participant dependence, robust variance estimates clustered by participant ID were used, and the Cox results were complemented by non-parametric Kaplan–Meier summaries, RMST estimates, and paired within-subject non-parametric tests.

#### 2.4.2. Primary Survival Analysis

The primary analysis compared neurologic and functional domains based on TTFI. Time-to-event distributions were estimated using Kaplan–Meier survival curves, with survival defined as the probability of not yet reaching MCID-defined improvement. Kaplan–Meier curves were used to visualize time-to-event patterns. Restricted mean survival time (RMST) estimates were additionally reported to provide a clinically interpretable summary of the average event-free time over the observation window. RMST was used as a complementary descriptive measure of recovery timing, whereas formal statistical inference regarding differences between groups was based on Cox proportional hazards models. For each comparison, a dynamic restricted mean survival time (RMST) horizon (τ) was set as the minimum of the maximum observed follow-up times between the two groups, ensuring identical observation windows. Differences between domains were formally tested using Cox proportional hazards regression models with cluster-robust variance estimation at the participant level to account for within-subject dependence caused by multiple scale contributions to composite outcomes. In each Cox proportional hazards model, the only predictor variable was a binary indicator representing the comparison group under study; no additional clinical covariates were included. Robust variance estimates clustered by participant ID were used to account for within-participant dependence. Hazard ratios (HRs) with 95% confidence intervals (CIs) were calculated with the functional domain as the reference category. The proportional hazards assumption was tested using Schoenfeld residual-based tests. Cox models were fitted only when both comparison groups had at least two events; otherwise, Cox results were reported as non-estimable, and interpretation was based on descriptive and paired analyses. Additionally, responder analyses were conducted for the primary comparison. For each group, the proportion of participants reaching MCID by prespecified time points (30, 60, 60, 90, 120, and 150 days) was calculated, with time points less than or equal to the dynamic τ. Responder proportions were estimated using exact binomial confidence intervals.

#### 2.4.3. Secondary and Intra-Domain Analyses

A secondary domain-level analysis compared GRASSP neurologic components (strength and Sensation) to GRASSP functional components (GRASSP_PA and GRASSP_PP) using the same survival framework, which included Kaplan–Meier estimation, dynamic RMST calculation, Cox regression with cluster-robust variance when estimable, and responder analysis. In addition to domain-level composites, intra-domain analyses were utilized to evaluate the timing of recovery across different scales within the same clinical construct. In the neurologic domain, pairwise comparisons were carried out between UEMS, GRASSP Strength, and GRASSP Sensation. In the functional domain, comparisons were made between GRASSP-PA, GRASSP-PP, and SCIM.

Each intra-domain comparison included a survival analysis (Kaplan–Meier estimation and Cox proportional hazards regression with cluster-robust variance, when estimable), as well as a paired within-subject TTFI analysis. Paired analyses reported the median difference in TTFI with interquartile range and assessed directionality with a binomial sign test, followed by Wilcoxon signed-rank testing when at least ten non-zero differences were available.

#### 2.4.4. Pairwise Inter-Scale Comparisons

Pre-specified pairwise comparisons of individual scales were performed to investigate recovery timing across neurologic and functional constructs, including comparisons of UEMS, SCIM, and GRASSP subscales. Each pairwise comparison included a survival analysis (Kaplan–Meier estimation, dynamic RMST, and Cox regression when estimable), as well as a paired within-subjects analysis. Cox regression results were only reported when both groups had at least two events; otherwise, comparisons were described with RMST and paired statistics. To account for multiple testing, false discovery rate correction using the Benjamini–Hochberg procedure was applied to inferential *p*-values arising from the pairwise Cox regression tests and paired within-subject comparisons. Kaplan–Meier curves and restricted mean survival time (RMST) estimates were treated as descriptive summaries and were not included in the multiplicity-adjustment procedure.

#### 2.4.5. AIS-Stratified Analyses

To assess whether recovery timing differed by injury severity, the primary domain-level analysis was repeated separately within AIS C and AIS D subgroups. All analytic steps were replicated within each subgroup.

#### 2.4.6. Exploratory Dominant vs. Non-Dominant Limb Analyses

Exploratory analyses examined potential differences in recovery timing between dominant and non-dominant limbs for limb-specific measures only (UEMS and GRASSP components). SCIM was excluded as it is not limb-specific. For each measure, dominant and non-dominant TTFI were compared using Kaplan–Meier estimation, dynamic RMST calculation, Cox regression when estimable, and paired within-subject analyses. These analyses were considered exploratory and hypothesis-generating.

All analyses were conducted using Python 3.11.9. Survival analyses were implemented using the lifelines library, and non-parametric statistical tests were performed using SciPy 1.15.3. Multiple-testing correction was implemented using the statsmodels package.

#### 2.4.7. Sample Size

No formal a priori sample size calculation was performed because the study was designed as an exploratory observational analysis based on the consecutive cohort of patients admitted to our Spinal Cord Unit during the study period. To mitigate the limited statistical power, we combined survival analysis (Kaplan–Meier and RMST) with False Discovery Rate (FDR) corrections, interpreting subgroup and pairwise comparisons as hypothesis-generating observations. In survival analysis, the effective sample size depends primarily on the number of observed events and the primary inferential comparison. Accordingly, the present study was intended to explore temporal recovery patterns rather than provide definitive subgroup or etiologic comparisons. To improve robustness, the analysis combined complementary descriptive and inferential approaches, and subgroup findings were interpreted as exploratory.

### 2.5. Data Privacy and GDPR Compliance

This study was conducted in full compliance with the General Data Protection Regulation (GDPR) and all applicable data protection regulations. Personal data were collected, managed, and stored following strict measures to guarantee confidentiality and data security. To safeguard participants’ privacy, all personally identifiable information was anonymized prior to data analysis.

## 3. Results

Twenty-one subjects were included in the analyses. According to clinical history and patient interviews all participants were fully independent in activities of daily living prior to the SCI. The total number of rehabilitation sessions (including both conventional physiotherapy and occupational therapy) was 142.76 ± 46.94. Demographic and neurological data for the total cohort on individuals with SCI are reported in Table 1, while additional baseline clinical information are detailed in Table 2.

Four participants completed fewer assessments than the maximum planned observation window due to discharge-related factors. Participant I-SCI16 was discharged earlier than scheduled for family-related organizational reasons requiring an earlier return home. Participants I-SCI15, I-SCI17, and I-SCI18 were transferred to residential care facilities because adequate home support was not available. None of these cases were related to medical complications or clinical deterioration.

Across individual scales, MCID was reached by 19 of 21 subjects (90.5%) for the UEMS (median TTFI 28.0 days [IQR 14.0–45.0]), by 15 of 21 subjects (71.4%) for SCIM (median TTFI 20.0 days [IQR 14.0–41.5]), by 19 of 21 subjects (90.5%) for GRASSP Strength (median TTFI 28.0 days [IQR 14.5–45.0]), by 7 of 21 subjects (33.3%) for GRASSP Sensation (median TTFI 43.0 days [IQR 27.0–70.5]), by 12 of 21 subjects (57.1%) for GRASSP-PA (median TTFI 49.5 days [IQR 27.8–64.0]), and by 16 of 21 subjects (76.2%) for GRASSP-PP (median TTFI 28.5 days [IQR 14.0–50.0]).

### 3.1. Primary Analysis: Neurologic vs. Functional Domains

**Figure 2:**
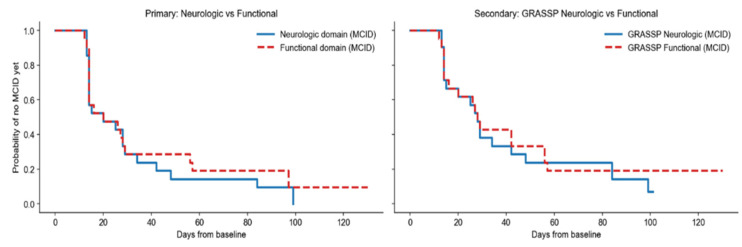
**Left panel**: Primary comparison between the Neurologic domain (earliest Minimal Clinically Important Difference [MCID] across Upper Extremity Motor Score [UEMS], GRASSP Strength, and GRASSP Sensation) and the Functional domain (earliest MCID across Spinal Cord Independence Measure III [SCIM], GRASSP Ability [GRASSP-PA], and GRASSP Performance [GRASSP-PP]). **Right panel**: Secondary comparison between GRASSP Neurologic (earliest MCID across GRASSP Strength and Sensation) and GRASSP Functional (earliest MCID across GRASSP-PA and GRASSP-PP). The y-axis represents the probability of not yet having achieved the MCID, while the *x*-axis represents days from baseline assessment. Each downward step in the curve corresponds to one or more participants reaching the MCID at that time point. Curves that decline earlier indicate faster achievement of clinically meaningful improvement. Overlapping curves indicate similar temporal recovery profiles, whereas persistent separation suggests differences in the timing of improvement between domains. Participants who did not reach MCID during follow-up were censored at their last available observation.

Kaplan–Meier curves showed largely overlapping survival distributions between neurologic and functional domains (Figure 2). The dynamically defined restricted mean survival time horizon was τ = 99.0 days. RMST was 32.1 days for the neurologic domain and 36.9 days for the functional domain (RMST difference −4.8 days, neurologic minus functional). Cox proportional hazards regression with clustering by subject yielded a hazard ratio of 1.17 (95% CI 0.64–2.15; *p* = 0.61), with no evidence of violation of the proportional hazards assumption (*p* = 0.77) (Table 2).

Paired within-subject analysis including all 21 subjects showed a median paired TTFI difference (functional minus neurologic) of 0.0 days (IQR −13.0 to 14.0). Neither the sign test (*p* = 0.80) nor the Wilcoxon signed-rank test (*p* = 0.57) was statistically significant (Table 2).

### 3.2. Secondary Analysis: GRASSP Neurologic vs. GRASSP Functional Components

**Figure 3:**
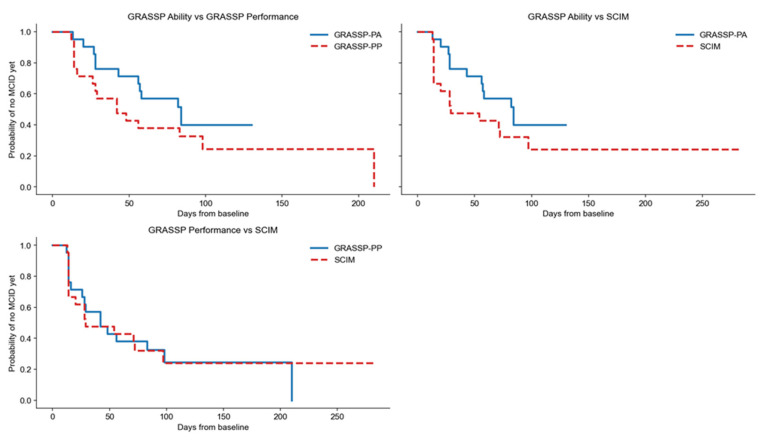
Intra-domain analysis: functional. Kaplan–Meier curves showing time to first minimal clinically important difference (MCID) for GRASSP Ability (GRASSP-PA) versus GRASSP Performance (GRASSP-PP) (**top left**), GRASSP-PA versus Spinal Cord Independence Measure (SCIM) (**top right**), and GRASSP-PP versus SCIM (**bottom**). The *y*-axis represents the probability of not yet reaching MCID; downward steps indicate clinically meaningful improvement. Earlier declines reflect faster recovery. GRASSP-PP shows a tendency toward earlier improvement compared with GRASSP-PA, whereas SCIM largely overlaps with both GRASSP functional subscales, indicating similar timing of functional gains.

The GRASSP neurologic composite (earliest MCID across GRASSP Strength and Sensation) was compared with the GRASSP functional composite (earliest MCID across Prehension Ability and Performance). Kaplan–Meier curves were estimated with a dynamically defined τ of 101.0 days (Figure 3).

RMST was 40.3 days for the GRASSP neurologic composite and 42.1 days for the GRASSP functional composite (RMST difference −1.8 days). Cox regression yielded a HR of 1.12 (95% CI 0.57–2.21; *p* = 0.73), with no evidence of proportional hazards violation (*p* = 0.38) (Table 3).

Paired analysis showed a median paired difference (functional minus neurologic) of 0.0 days (IQR −14.0 to 14.0), with non-significant sign (*p* = 0.80) and Wilcoxon tests (*p* = 0.96) (Table 3).

### 3.3. Intra-Domain Analyses

Within the neurologic domain, comparisons between UEMS and GRASSP Strength showed similar TTFI distributions with overlapping Kaplan–Meier curves (RMST difference ≈ 0; HR ≈ 1, *p* > 0.05) (Figure 3), and paired analyses did not show significant differences (median paired difference ≈ 0; Wilcoxon *p* > 0.05) (Table 3). Conversely, comparisons involving GRASSP Sensation demonstrated substantially delayed MCID achievement. In the comparison between UEMS and GRASSP Sensation, RMST was significantly longer for Sensation (RMST difference +71.0 days), with a significantly reduced hazard of MCID achievement (HR 0.16, 95% CI 0.07–0.33; *p* < 0.001; FDR-adjusted *p* < 0.001). Paired analysis showed a median paired difference of 42.0 days (IQR 29.0–71.0), with a significant Wilcoxon test (raw *p* < 0.001; FDR-adjusted *p* = 0.002) (Table 4).

Similarly, GRASSP Strength vs. GRASSP Sensation showed delayed sensory recovery (RMST difference +71.2 days; HR 0.17, 95% CI 0.08–0.37; *p* < 0.001; FDR-adjusted *p* < 0.001), with a median paired difference of 43.0 days (IQR 29.0–71.0; Wilcoxon raw *p* < 0.001, FDR-adjusted *p* = 0.002) (Table 4).

### 3.4. Pairwise Inter-Scale Comparisons

Fifteen pre-specified pairwise inter-scale comparisons were performed. False discovery rate (FDR) correction was applied to all pairwise survival and paired within-subject analyses.

Comparisons between GRASSP Strength and GRASSP-PA showed earlier MCID achievement for strength, with a restricted mean survival time (RMST) of 42.429 days for GRASSP Strength and 70.400 days for GRASSP-P (RMST difference [PA − Strength] = +27.971 days). Cox regression indicated a significantly higher hazard of MCID achievement for strength (HR = 0.390, 95% CI 0.210–0.750; *p* = 0.004; FDR-adjusted *p* = 0.017). Paired analysis supported this finding, with strength reaching MCID earlier than GRASSP-PA in most of the subjects (median paired difference = 28 days [IQR 14–56]; Wilcoxon *p* = 0.013; FDR-adjusted *p* = 0.030).

Conversely, the comparison between GRASSP Strength and GRASSP-PP showed earlier MCID achievement for strength, but this difference did not reach statistical significance. RMST was 42.429 days for GRASSP Strength and 54.157 days for GRASSP-PP (RMST difference = +11.728 days). Cox regression was non-significant (HR = 0.660, 95% CI 0.350–1.230; *p* = 0.188; FDR-adjusted *p* = 0.257), and paired analysis showed a small and non-significant median paired difference of 13 days (IQR −13 to 28; Wilcoxon *p* = 0.245; FDR-adjusted *p* = 0.312). Although RMST differences and paired medians consistently favored earlier recovery of strength relative to GRASSP-PP, the lack of statistical significance suggests either a smaller effect size or limited power, and this comparison should be interpreted as exploratory.

All comparisons involving GRASSP Sensation consistently demonstrated significantly delayed MCID achievement relative to motor-based and functional measures. In the comparison between GRASSP Sensation and GRASSP-PA, RMST was longer for Sensation (100.953 vs. 82.000 days; RMST difference [PA − Sensation] = −18.953 days). Cox regression indicated a non-significant trend toward slower recovery for Sensation (HR = 2.133, 95% CI 0.836–5.443; *p* = 0.113; FDR-adjusted *p* = 0.184), and paired analysis showed no significant difference (median paired difference = 0 days [IQR −97 to 14]; Wilcoxon *p* = 0.197; FDR-adjusted *p* = 0.296). Conversely, the comparison between GRASSP Sensation and GRASSP-PP demonstrated a statistically significant delay in sensory recovery. RMST was 138.538 days for Sensation and 80.850 days for GRASSP-PP (RMST difference [PP − Sensation] = −57.688 days). Cox regression showed a significantly lower hazard of MCID achievement for Sensation (HR = 3.289, 95% CI 1.345–8.046; *p* = 0.009; FDR-adjusted *p* = 0.029). Paired analysis confirmed this finding, with a median paired difference of 1 day favoring earlier GRASSP-PP improvement (IQR −56 to 14; Wilcoxon *p* = 0.007; FDR-adjusted *p* = 0.029).

**Figure 4:**
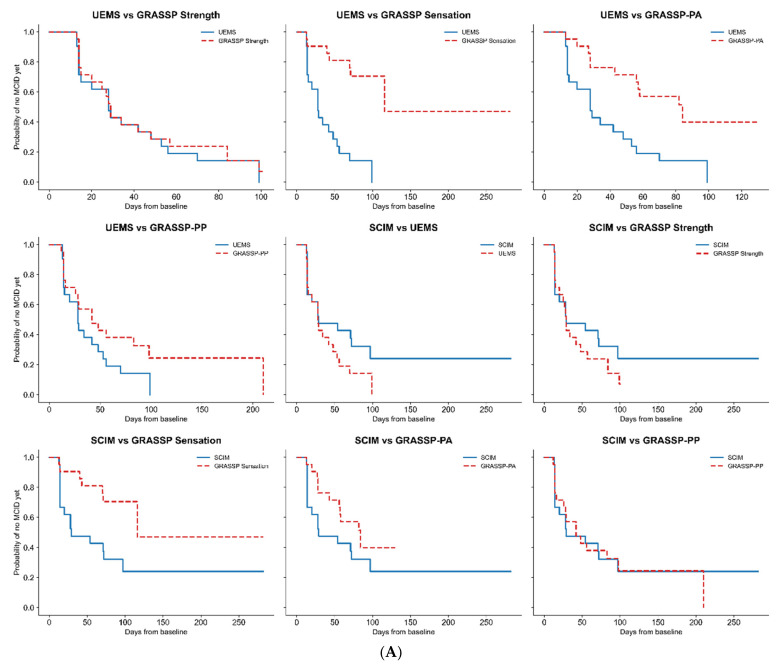

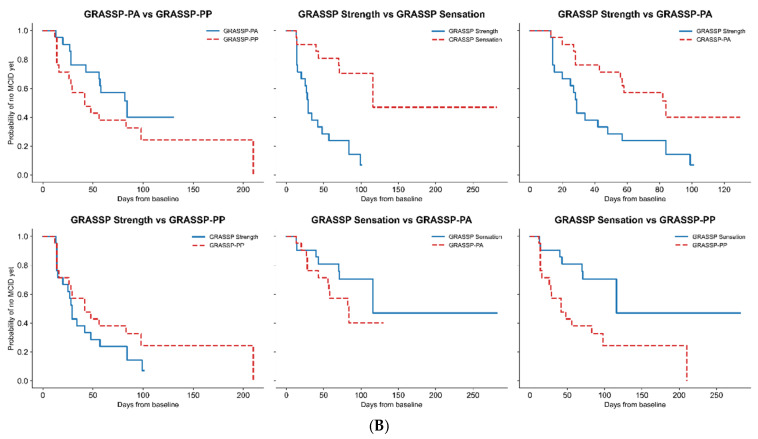
(**A**) Pairwise inter-domain comparisons involving UEMS and SCIM. Kaplan–Meier survival curves show the probability of not yet reaching the Minimal Clinically Important Difference (MCID) over time for pairwise comparisons between the global neurological (UEMS) and functional (SCIM) scales and the GRASSP subscales. Each panel contrasts two outcome measures (solid vs. dashed line). A steeper decline indicates earlier achievement of MCID. Visual separation reflects differences in recovery timing, with motor measures (UEMS, GRASSP Strength) generally improving earlier than sensory measures (GRASSP Sensation), while functional measures (GRASSP-PA, GRASSP-PP, SCIM) show intermediate and partially overlapping trajectories. Formal between-scale comparisons are quantified using restricted mean survival time (RMST), Cox proportional hazards models, and paired within-subject analyses (see Table 5). (**B**) Pairwise comparisons among GRASSP components. Kaplan–Meier survival curves show the probability of not yet reaching the Minimal Clinically Important Difference (MCID) over time for pairwise comparisons among GRASSP neurologic and functional subscales. Each panel contrasts two outcome measures (solid vs. dashed line). A steeper decline indicates earlier achievement of MCID. Differences between curves illustrate the relative timing of recovery across GRASSP components, highlighting the earlier improvement typically observed in motor-related measures compared with sensory or functional components. Formal between-scale comparisons are quantified using restricted mean survival time (RMST), Cox proportional hazards models, and paired within-subject analyses (see Table 5).

Comparisons involving SCIM did not demonstrate statistically significant differences after FDR correction, despite some nominal trends. For example, SCIM vs. GRASSP Sensation showed a longer RMST for Sensation (RMST difference = +76.737 days), with a significant unadjusted Cox *p*-value (*p* = 0.011) that did not remain significant after FDR correction (*p* = 0.029). Paired analysis similarly showed earlier SCIM improvement (median paired difference = 28 days [IQR 0–78]), but this finding should be interpreted cautiously given the multiplicity of comparisons (Figure 4A,B, Table 5).

### 3.5. AIS-Stratified Primary Analysis

**Figure 5:**
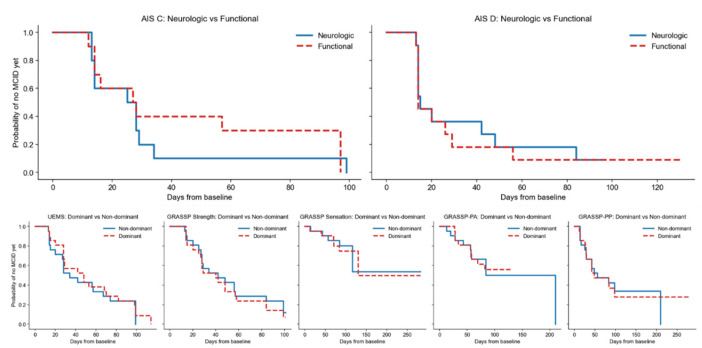
Top panels show Kaplan–Meier curves comparing neurologic and functional domains in individuals with AIS C (**left**) and AIS D (**right**) cervical spinal cord injury. Bottom panels display dominant versus non-dominant limb comparisons for limb-specific measures (Upper Extremity Motor Score [UEMS], GRASSP Strength, GRASSP Sensation, GRASSP Ability [GRASSP-PA], and GRASSP Performance [GRASSP-PP]). The y-axis represents the probability of not yet reaching the minimal clinically important difference (MCID), and the x-axis represents days from baseline. Overlapping curves indicate similar timing of clinically meaningful improvement between groups; group separation reflects earlier MCID achievement. No statistically significant differences were observed between dominant and non-dominant limbs or between neurologic and functional domains within AIS strata after appropriate statistical testing.

In AIS C subjects (*n* = 10), τ was 97.0 days, with RMST 29.5 days for the neurologic domain and 45.9 days for the functional domain (RMST difference −16.4 days; HR 1.36, 95% CI 0.66–2.84; *p* = 0.41). Paired analysis showed a median paired difference of −2.0 days (IQR −2.0 to 13.0; sign test *p* = 1.00) (Figure 5, Table 5).

In AIS D subjects (*n* = 11), τ was 97.0 days, with RMST 34.1 vs. 28.3 days (RMST difference +5.8 days; HR 0.87, 95% CI 0.36–2.09; *p* = 0.76). Paired analysis showed a median paired difference of 0.0 days (IQR −1.0 to 5.0; sign test *p* = 1.00) (Figure 5, Table 6).

### 3.6. Exploratory Dominant vs. Non-Dominant Limb Analyses

Across limb-specific measures, dominant vs. non-dominant comparisons showed overlapping Kaplan–Meier curves, RMST differences close to zero, hazard ratios near unity, and non-significant paired analyses (all *p* > 0.05) (Figure 5, Table 7).

## 4. Discussion

The aim of this prospective longitudinal open-cohort study was to evaluate the temporal relationship between neurological and functional recovery in individuals with tetraplegia using complementary clinical scales. Specifically, we examined neurological and functional recovery in individuals with sensory-motor incomplete tetraplegia through repeated assessments over time from admission to the Spinal Cord Unit [18,19,23]. The findings of this study describe the temporal dynamics of recovery observed during inpatient rehabilitation rather than the effect of specific rehabilitation components. The rehabilitation setting provides clinically relevant context, but the study was not designed to compare rehabilitation strategies or support causal inference regarding therapeutic interventions. All statistical analyses were conducted from an individual-centered perspective [28], using the MID, MCD or MCID as the primary interpretative framework. This approach was adopted to move beyond statistically detectable changes and to focus instead on improvements that are likely to be perceived as meaningful by individuals in their daily experience. By interpreting neurological and functional outcomes through the lens of MCID, the present findings aim to reflect clinically relevant recovery patterns rather than purely metric changes, thereby enhancing their translational value for rehabilitation practice.

Given the severe consequences for individual independence, quality of life, healthcare demands, and socioeconomic burden, there is a strong need for reliable tools to assess and categorize upper limb function in tetraplegia [2,3]. Existing assessments, such as the Van Lieshout Test [29] and the Capability of Upper Extremity Test [30] provide important information on overall arm and hand use but are not designed to capture specific changes in sensory and motor impairments. Similarly, global outcome measures such as the SCIM, while clinically meaningful for assessing independence in activities of daily living [19], does not provide information on underlying neurological. As a result, improvements in SCIM scores may reflect a combination of neural recovery, compensatory strategies, and contextual factors such as training, motivation, or task execution (e.g., bimanual or compensatory movements), limiting their ability to isolate mechanisms of recovery [31,32]. In contrast, outcome measures designed to detect neurological deficits such as the UEMS have demonstrated value for diagnosis and prognosis of SCI [33]. Clinical experience shows that recovery of upper limb function is highly variable, and that integrating neurological and functional assessment in acute cervical SCI may provide a more comprehensive framework to guide strategies [13,34].

The present findings indicate a coordinated temporal relationship between neurological and functional recovery in individuals with cervical SCI undergoing rehabilitation. When outcome measures [18,19,23] were analyzed by domains, neurological and functional improvements showed largely overlapping temporal trajectories, with no statistically significant difference in the timing of first clinically meaningful improvement. This finding suggests that, when recovery is interpreted using MCID-based thresholds, functional gains may emerge in parallel with neurological changes rather than strictly following them. This suggests that clinically meaningful neurological and functional gains may emerge concurrently at a macro level, even when their underlying components follow different temporal dynamics. This observation is particularly relevant because it emerged in a cohort of individuals with SCI receiving a treatment-as-usual rehabilitation program. The intensity and structure of rehabilitation sessions were broadly similar across participants, reflecting real-world clinical practice rather than a highly controlled experimental setting. Although the overall duration (i.e., length of stay in the Spinal Cord Unit) of rehabilitation differed among individuals, participants were exposed to a comparable therapeutic framework, including physiotherapy, occupational therapy and functional training with no robotic devices targeting both upper and lower limbs. From a clinical perspective, these general findings about domains suggest that functional use of the upper limb, such as effective grasping, object manipulation, and independence in ADLs, likely depends on the availability of an adequate neurological substrate, even though neurological and functional improvements may manifest concurrently when evaluated using clinically meaningful thresholds. Neurological recovery therefore appears to provide a physiological foundation that supports, rather than strictly precedes, functional improvement. This temporal interpretation has important implications for clinical reasoning and goal setting in rehabilitation. Early improvements in neurological measures should not be interpreted as an immediate failure to achieve functional gains, but rather as part of a physiological and functional continuum in which neurological recovery supports the emergence of functional abilities. Recognizing this relationship may help clinicians better interpret outcome measures over time, tailor rehabilitation goals according to recovery phase, and manage expectations during the early stages of rehabilitation. Importantly, the main findings were not based on a single analytical approach. Rather, temporal patterns were assessed using complementary methods, including Kaplan–Meier curves, RMST summaries, Cox proportional hazards models, responder analyses, and paired within-subject comparisons. The concordance in direction and interpretation across these approaches, particularly the earlier recovery of motor strength relative to sensory and dexterity-related outcomes, supports the findings’ robustness despite the small sample size.

A more detailed analysis within each domain provided further insights. Within the neurological domain, a consistent temporal pattern emerged, with motor recovery of the upper limb preceding sensory improvement. Specifically, a significant increase in UEMSs was observed before detectable changes in sensory outcomes. This motor improvement was captured by both the UEMS and the motor component of the GRASSP, indicating a convergent ability of these two measures to identify early neurological changes. The concurrent improvement detected by UEMS and GRASSP motor subscores is particularly relevant, as these tools assess partially overlapping but not identical sets of upper limb muscles using different scoring approaches. The fact that both measures consistently detected motor recovery supports their clinical robustness and sensitivity in monitoring early neurological changes in individuals with cervical SCI. Despite methodological differences, both scales appear to reliably capture improvements in voluntary motor output of the upper extremities during the early phases of rehabilitation. Notably, these motor improvements systematically preceded gains in sensory function. This pattern suggests that recovery of voluntary muscle activation may reach clinically meaningful thresholds earlier than measurable changes in sensory pathways, at least within the timeframe of inpatient rehabilitation. From a neurorehabilitation perspective, this finding is clinically meaningful, as it highlights that early neurological recovery may preferentially manifest through motor rather than sensory channels.

For clinicians, this interpretation of results has practical implications. Early improvements in motor scores should be recognized as meaningful indicators of neurological recovery, even in the absence of parallel sensory changes. Moreover, the combined use of UEMS and GRASSP motor subscores may offer complementary and confirmatory information, strengthening clinical confidence in the detection of early motor recovery while acknowledging that sensory restitution may follow a different and potentially slower trajectory. These findings align with the principles of Activity-Based Rehabilitation (ABR) [35,36], suggesting a staged approach to therapy. The observation that motor strength improvements precede fine dexterity suggests that early ABR protocols should prioritize high-intensity, gross motor activation to build the necessary neurological substrate. Subsequently, as sensory recovery emerges, typically on a slower trajectory, rehabilitation can effectively shift toward task-specific training and sensorimotor integration to refine prehension and functional independence. In addition, when the temporal evolution of motor and functional outcomes was examined more closely, upper extremity motor recovery assessed by the UEMS tended to precede or partially overlap with early improvements in gross upper limb ability. Pairwise analyses showed that clinically meaningful gains in GRASSP Strength generally occurred earlier than improvements in both prehension ability and prehension performance, suggesting that recovery of voluntary force production supports, but does not immediately translate into, functional hand use. In contrast, more complex aspects of upper limb function—such as fine motor skills, object manipulation, and functional independence in daily activities—emerged later in the rehabilitation process. Tasks requiring refined dexterity, precise finger coordination, and sensorimotor integration (e.g., manipulating small objects, inserting a key into a lock, or performance in fine motor assessments) appeared to require a longer recovery trajectory. These higher-level functional abilities appeared to benefit from sensory recovery, although this interpretation should be considered hypothesis-generating and requires confirmation in studies specifically designed to evaluate rehabilitation strategies.

Taken together, these findings indicate that motor strength, sensory recovery, and functional independence follow distinct temporal trajectories. While motor strength and gross ability may recover in parallel at an early stage, the achievement of fine motor control and functional independence appears to depend on the combined contribution of both motor and sensory systems. This reinforces the concept that meaningful functional recovery of the upper limb requires not only sufficient muscle strength but also adequate sensory feedback, particularly when complex and precise motor tasks are involved.

Although the present study was not designed to investigate underlying neurophysiological mechanisms, the observed temporal patterns may be interpreted in light of existing evidence on SCI recovery. To better contextualize the recovery patterns observed in the present study, it is important to consider the complex pathophysiology of SCI, which involves both a primary insult and a secondary cascade of biological processes triggered by the initial damage [37]. While the primary injury is largely irreversible and may lead to structural changes such as spinal cord atrophy [38], the secondary injury phase represents an important target for therapeutic interventions. Existing evidence indicates that secondary injury involves the immune system, nervous system and vascular system including hemorrhage, ischemia, oxidative stress, inflammatory reaction, neural cell death, demyelination, scar formation [37]. At the cellular level, ion channels regulating neuronal excitability in dorsal root ganglion neurons, such as TWIK-related spinal cord K+ channels (TRESK), have been shown to modulate sensory neuron activity and may participate in inflammatory processes following injury. Although their precise role in SCI recovery is not yet fully understood, these mechanisms may contribute to changes in sensory processing during the recovery phase [39].

Functional recovery largely reflects adaptive reorganization within residual neural networks. Neural plasticity may occur through compensatory mechanisms at both spinal and supraspinal levels, including cortical reorganization, synaptic plasticity, and sprouting of spared axons [38]. SCI has been shown to induce structural and functional changes within the sensorimotor cortex and reorganization of both motor and sensory areas. The degree of cortical reorganization has been reported to correlate with the severity of SCI and the resulting level of disability, although the cortical and subcortical mechanisms underlying sensory reorganization remain incompletely understood [38]. Interestingly, neural plasticity after SCI may also involve thalamic structures. Imaging studies suggest that regions corresponding to sensory thalamic nuclei may undergo structural changes related to sensory function supporting the hypothesis that long-term thalamic reorganization may occur following SCI, alongside the reorganization of sensorimotor cortical networks described in individuals with cervical SCI. Such central reorganization processes may also contribute to differences in the temporal trajectories of motor and sensory recovery, although the mechanisms underlying these dynamics remain incompletely understood [40]. These mechanisms provide a plausible framework for interpreting the observed temporal dissociation between motor and sensory recovery, although they were not directly assessed in the present study. Within the spinal cord, spared tissue and partial preservation of sensorimotor pathways, including corticospinal projections mediated through spinal interneurons, may contribute to the formation of alternative neural circuits supporting functional recovery. Importantly, sensory function is not simply a passive by-product of motor rehabilitation but may undergo independent forms of plastic reorganization [41]. Experimental and clinical evidence suggests that proprioceptive and mechanoreceptive circuits can remodel below the level of injury, potentially providing alternative pathways for the transmission of afferent information [42].

Longitudinal studies have shown that motor recovery follows a relatively well-defined temporal trajectory after SCI [43]. The majority of motor recovery occurs within the first six to nine months after injury, with the most rapid improvements typically observed during the first three months [13,43]. Individuals with AIS D injuries often show smaller changes in motor scores due to a ceiling effect but achieve the highest final motor performance. In contrast, the temporal dynamics of sensory recovery remain less well characterized. Compared with motor outcomes, relatively few studies have specifically investigated sensory recovery profiles after SCI. Available evidence suggests that changes in pinprick and light-touch scores in individuals with tetraplegia are highly variable over time and generally smaller in magnitude compared with motor recovery. Indeed, longitudinal analyses indicate that sensory deficits often show limited improvement over time following cervical SCI [13]. Consequently, the relative temporal relationship between motor and sensory recovery after cervical incomplete SCI remains uncertain. The findings of the present study contribute to this ongoing discussion by providing longitudinal observations of both neurological and functional recovery trajectories during inpatient rehabilitation.

Furthermore, when comparing dominant and non-dominant upper limbs, the overall patterns of neurological and functional recovery appeared largely similar. The temporal sequence of motor, sensory, and functional improvements followed comparable trajectories in both limbs. Although a slight anticipation of recovery was observed in the dominant hand for prehension ability and prehension performance, this difference was subtle and did not reach clinical or statistical significance. This finding suggests that recovery mechanisms are not strongly lateralized and underscores the importance of directing rehabilitative attention to both upper limbs, rather than prioritizing the dominant hand. From a clinical standpoint, focusing exclusively on the dominant side may risk underestimating the functional potential of the non-dominant upper limb, which plays a crucial role in bimanual tasks and overall independence in activities of daily living.

Distinct recovery patterns also emerged when stratifying individuals by neurological status (i.e., AIS classification). Differences between AIS C and AIS D individuals can be interpreted in light of their baseline neurological profiles, particularly the number of key muscles presenting with a motor score greater than or equal to 3 at neurological examination. This fundamental distinction implies a different starting point in terms of voluntary motor control and functional reserve. In individuals classified as AIS C, motor recovery tended to precede functional improvement, suggesting that gains in muscle strength may represent an important facilitator for subsequent functional achievements. In contrast, ASIA D individuals showed a more parallel evolution of motor and functional recovery, likely reflecting higher baseline strength levels that allow functional gains to emerge earlier and more concurrently with motor improvements. In exploratory AIS-stratified analyses, AIS C participants showed a numerical tendency for neurological recovery to precede functional recovery, whereas AIS D participants showed a more parallel temporal pattern. One could argue that in AIS C individuals, treatment strategies may benefit from an early and intensive focus on motor recovery, with physiotherapy playing a central role in enhancing strength and voluntary activation. Conversely, in AIS D individuals, where motor capacity is often already present, rehabilitation may more effectively emphasize functional training and occupational therapy, targeting task-specific skills, dexterity, and independence in ADLs. However, these subgroup differences were not statistically significant and should therefore be interpreted cautiously given the limited sample size. These findings should be considered hypothesis-generating and require confirmation in larger, adequately powered cohorts before informing personalized rehabilitation strategies.

Furthermore, a previous review [44] showed that the majority of AIS conversion and motor recovery occurs within the first 6–9 months after injury, with the most rapid rate of motor recovery observed during the first three months. Our results are consistent with the early timing of recovery; however, they also indicate that, although sensory recovery appears to begin later, this delay may be functionally compensated by early motor recovery occurring during the initial phase of rehabilitation. Considering that the timing of baseline neurological examinations may be associated with the extent of motor recovery in cervical AIS B, C, and D injuries [45], a strength of our study is the early timing of the assessment, which was performed within the first days after admission to the rehabilitation facility. In this context, our results suggest that the initial evaluation could be integrated with both neurological and functional assessments, potentially improving the assessment of strength and sensory function by allowing the early identification of measures that are more responsive and clinically meaningful as tools for guiding tailored rehabilitative treatment.

### Study Limitations

Some limitations of this study should be acknowledged. First, although all individuals with cervical SCI admitted to the Spinal Cord Unit during the study period were included, the overall sample size remained relatively limited. This limitation is particularly relevant given the use of time-to-event methods and multiple pairwise comparisons, which, despite appropriate false discovery rate correction, may reduce statistical power for detecting modest between-scale differences. As a consequence, stratified analyses based on age were not feasible. This represents a relevant limitation, as age-related differences in recovery trajectories may exist. While neurological recovery potential appears to be preserved across age groups, the translation of neurological improvements into functional performance may vary with increasing age [46]. Future studies should therefore investigate whether similar recovery patterns are observed in substantially older populations. Second, not all individuals completed the full set of assessments at every time point. This limitation reflects the inherent constraints of routine inpatient rehabilitation, including variations in medical stability, fatigue, and length of stay [47]. From a statistical standpoint, this resulted in right-censoring and unbalanced follow-up across measures, which was explicitly handled using survival analysis techniques [48,49] rather than complete-case or imputation-based approaches. Although this approach allows robust use of all available information, estimates of restricted mean survival time and hazard ratios are conditional on the observed follow-up window and may be sensitive to the timing of assessments. Third, time-to-first improvement was defined from the first available rehabilitation assessment rather than from injury onset, because the primary aim was to characterize recovery timing from rehabilitation entry. Although this choice reflects the clinical rehabilitation setting, it also means that participants entered observation at different post-injury stages, which may have affected comparability of recovery timing across individuals.

Fourth, our cohort reflects the clinical heterogeneity of a real-world Spinal Cord Unit, including both traumatic and non-traumatic etiologies. This heterogeneity may limit generalizability across etiologic subgroups; however, the primary analyses were based on within-patient time-to-first-improvement relative to each individual’s own baseline, and were therefore intended to characterize temporal recovery relationships rather than support comparisons across etiologic categories. Nevertheless, the present sample size did not permit etiology-specific analyses, which should be addressed in future larger studies.

Fifth, the thresholds for MDC, MID, and MCID were selected based on the most current available literature for each scale. However, the reliance on these pre-established values represents a potential limitation, as they may not fully capture the individual variability in perceived meaningful change across different stages of recovery [50]. In addition, the use of distinct MCID thresholds across scales may influence the relative timing of detected improvements and should be considered when interpreting cross-scale temporal comparisons.

Finally, although rehabilitation exposure was relatively homogeneous across participants and reflects treatment-as-usual clinical practice, the study was not designed to isolate the effects of specific rehabilitation components. While the consistency of the therapeutic approach supports the interpretability and ecological validity of the observed recovery patterns, the lack of experimental control precludes causal attribution of these patterns to specific rehabilitation strategies. In addition, detailed quantification of rehabilitation content (e.g., relative emphasis on gross versus fine motor training) was not systematically available, and future studies using structured rehabilitation logs may help disentangle the contribution of neurological recovery and therapy content to functional outcomes.

Another limitation concerns the level of detail available for some baseline clinical variables. Although core demographic and neurological descriptors, comorbidities, surgical management, and pre-admission functional status were collected, additional factors such as standardized MRI-based measures of spinal cord compression and detailed pharmacological management during hospitalization were not available in a structured format for retrospective analysis. These variables could potentially influence recovery trajectories and should be considered in future studies integrating imaging biomarkers and treatment data.

## 5. Conclusions

The recovery patterns observed in this study offer several clinically relevant implications for the rehabilitation of individuals with cervical SCI. First, the evidence of a coordinated evolution of neurological and functional recovery, with overlapping timing at the domain level, supports a phase-specific approach to rehabilitation. During the early stages, when motor and sensory recovery are still emerging, rehabilitation programs may benefit from prioritizing interventions aimed at enhancing voluntary muscle activation, strength, and sensory input, rather than focusing exclusively on complex functional tasks. The finding that improvements in upper limb motor strength tend to precede or partially overlap with gains in gross prehension ability suggests that early rehabilitation should leverage this window by integrating strength-oriented physiotherapy with task-oriented activities targeting basic grasping and object stabilization. This combined approach may facilitate the translation of motor recovery into functional use of the upper limb without prematurely overloading individuals with tasks requiring refined dexterity and high sensorimotor integration. Conversely, the delayed emergence of fine motor control and functional independence highlights the importance of timing in occupational therapy interventions. Tasks requiring precision, manipulation of small objects, and fine finger coordination appear to benefit from the combined recovery of both motor and sensory systems. Therefore, introducing highly demanding fine motor tasks may be more effective once sensory recovery has reached a sufficient level, allowing individuals to fully benefit from such training. The similar recovery trajectories observed in dominant and non-dominant upper limbs further emphasize the need for bilateral rehabilitation strategies actively engaging both hands, to prevent the non-dominant limb from becoming functionally neglected. Finally, the different recovery patterns observed between ASIA C and ASIA D individuals suggest that rehabilitation strategies should be tailored according to neurological severity. Adopting severity-specific rehabilitation pathways may therefore enhance efficiency, optimize outcomes, and support more personalized rehabilitation planning.

## Figures and Tables

**Table 1:** Demographic and clinical characteristics of the study population.

	Sex	Age (y)	Aetiology	AIS	Neurological Level of Injury	Days Between SCI and T1 Assessment	Number of Assessments Performed
I-SCI1	M	39	Traumatic	D	C6	19	8
I-SCI2	F	85	Traumatic	D	C6	29	8
I-SCI3	M	58	Traumatic	C	C4	46	8
I-SCI4	M	37	Traumatic	D	C7	61	8
I-SCI5	M	47	Non-traumatic (Cervical degenerative Myelopathy)	C	C7	28	8
I-SCI6	M	33	Traumatic	D	C4	22	8
I-SCI7	M	69	Non-traumatic (ischemic)	D	C4	20	8
I-SCI8	M	52	Traumatic	D	C7	12	8
I-SCI9	M	30	Non-traumatic (tumor)	C	C7	13	8
I-SCI10	M	23	Traumatic	D	C7	25	8
I-SCI11	M	85	Traumatic	C	C7	86	8
I-SCI12	M	54	Traumatic	C	C7	12	8
I-SCI13	M	64	Non-traumatic (Cervical degenerative Myelopathy)	D	C4	99	8
I-SCI14	M	31	Traumatic	C	C7	39	8
I-SCI15	M	23	Traumatic	C	C7	164	5
I-SCI16	M	33	Traumatic	C	C6	26	5
I-SCI17	M	62	Traumatic	D	C7	21	5
I-SCI18	M	85	Traumatic	C	C4	32	6
I-SCI19	M	75	Traumatic	D	C7	13	8
I-SCI20	M	74	Non-traumatic (Cervical degenerative Myelopathy)	C	C7	178	8
I-SCI21	M	46	Non-traumatic (Cervical degenerative Myelopathy)	C	C7	43	8
**Mean ± SD/%**	**4.8% F**	**52.6 ± 20.8**	**71.4%** **Traumatic** **28.6%** **Non-Traumatic**	**52.4% C** **47.6% D**	**C4 23.8%** **C6 14.3%** **C7 61.9%**	**Median: 28**	**—**

I-SCI: individual with SCI; Y: years M: male, F: female.

**Table 2:** Additional baseline clinical information of the study population.

	Aetiology	Comorbidities or Previous Medical History	Pre-Existing Clinical Conditions in the Upper Limbs	Surgical Management
I-SCI1	T	-	-	Discectomy
I-SCI2	T	Bradycardia, hypertension, chronic bronchitis, urinary incontinence, hip replacement	Righ shoulder dislocation	-
I-SCI3	T	-	Surgery for supraspinatus muscle injury	Decompression and arthrodesis
I-SCI4	T	-	-	-
I-SCI5	NT	Diabetes	-	-
I-SCI6	T	-	-	-
I-SCI7	NT	Hypertension, dyslipidemia, diabetes, hyperuricemia, obstructive sleep apnea	-	-
I-SCI8	T	-	-	Decompression and arthrodesis
I-SCI9	NT	-	-	Microsurgical removal of spinal cord tumor, laminectomy and laminoplasty
I-SCI10	T	Brugada syndrome	-	Arthrodesis
I-SCI11	T	Chronic atrial fibrillation, hypertension, anemia, benign prostatic hypertrophy, diverticulosis, hearing loss	-	-
I-SCI12	T	Hypertension	-	Laminectomy
I-SCI13	NT	Chronic gastritis, benign prostatic hypertrophy	-	-
I-SCI14	T	-	-	Cervical Stabilization
I-SCI15	T	-	-	Dorsal stabilization and reduction in C7-D1 listhesis
I-SCI16	T	Ischemic heart disease, hypertension	-	-
I-SCI17	T	Hypertension, benign prostatic hypertrophy	-	Decompression and arthrodesis
I-SCI18	T	Hypertension, hypercholesterolemia, sleep apnea	-	Decompression and stabilization
I-SCI19	T	-	-	Somatectomy and stabilization
I-SCI20	NT	Previous pulmonary embolism	-	Somatectomy and arthrodesis
I-SCI21	NT	Hypertension, ischemic heart disease, diabetes, hypercholesterolemia	-	Laminectomy

I-SCI: individual with SCI; T: traumatic SCI; NT: non traumatic SCI.

**Table 3:** Neurologic vs. Functional domains results.

Comparison (A vs. B)	TAU RMST	RMST A	RMST B	RMST (B − A)	HR (95% CI)	*p* Cox	*p* PH
Neurologic vs. Functional domain	99	36.857	32.095	−4.762	1.175 (0.642—0.149)	0.602	0.764
GRASSP Neurologic vs. GRASSP Functional	101	42.095	40.333	−1.762	1.124 (0.564—0.197)	0.734	0.948

GRASSP = Graded Redefined Assessment of Strength, Sensation and Prehension; τ (TAU) = truncation time used for restricted mean survival time estimation; RMST = Restricted Mean Survival Time (lower values indicate earlier achievement of MCID); RMST (B − A) = difference in RMST between groups (negative values indicate earlier improvement in group B); HR = Hazard Ratio from Cox proportional hazards model (HR > 1 indicates higher probability of achieving MCID in group B); CI = Confidence Interval; *p* Cox = *p*-value from Cox regression; *p* PH = *p*-value testing the proportional hazards assumption.

**Table 4:** Intra-domain analyses.

Domain	Comparison (A vs. B)	TAU RMST	RMST A	RMST B	RMST (B − A)	HR (95% CI)	*p* Cox	*p* PH	*p* Cox FDR	Median TTFI B − A (IQR Range)	*p* Wilcoxon	*p* Wilcoxon FDR
Neurologic	UEMS vs. GRASSP Strength	99	39.524	42.286	2.762	0.894 (0.610–1.308)	0.563	0.491	0.610	0 (0–0)	na	na
	UEMS vs. GRASSP Sensation	99	39.524	82.396	42.872	0.156 (0.068–0.827)	0.000	0.827	0.000	42 (27–73)	0.002	0.010
	GRASSP Strength vs. GRASSP Sensation	101	42.429	83.805	41.377	0.174 (0.080–0.380)	0.000	0.708	0.000	41 (14–70)	0.001	0.010
Functional	SCIM vs. GRASSP-PA	130	58.985	82.000	23.015	0.597 (0.362–0.983)	0.043	0.018	0.092	0 (0–42)	0.049	0.099
	SCIM vs. GRASSP-PP	210	78.271	80.850	2.580	0.994 (0.598–1.653)	0.982	0.077	0.982	0 (−14–28)	0.959	0.959
	GRASSP-PA vs. GRASSP-PP	130	82.000	61.259	−20.741	1.639 (0.815–3.294)	0.166	0.229	0.239	0 (−14–−58	0.224	0.298

UEMS = Upper Extremity Motor Score; GRASSP = Graded Redefined Assessment of Strength, Sensation and Prehension; GRASSP-PA = GRASSP Ability; GRASSP-PP = GRASSP Performance; SCIM = Spinal Cord Independence Measure. TAU RMST denotes the restricted mean survival time horizon (τ). RMST A and RMST B represent the mean time to first minimal clinically important difference (MCID) within τ for groups A and B, respectively; RMST (B − A) indicates relative delay (positive values) or anticipation (negative values) of improvement for group B. HR = hazard ratio from Cox proportional hazards models (HR < 1 indicates slower MCID achievement for group B; HR > 1 faster achievement). *p* Cox reports the Cox model *p*-value; *p* PH assesses proportional hazards assumptions; FDR indicates false discovery rate–adjusted *p*-values for intra-domain comparisons. Median TTFI (B − A) with interquartile range (IQR) reflects paired within-subject differences; Wilcoxon *p*-values assess paired contrasts. All intra-domain comparisons were pre-specified; false discovery rate correction was applied to control for multiple testing in the context of limited sample size. False discovery rate (FDR) correction using the Benjamini–Hochberg procedure was applied to the inferential *p*-values reported for pairwise intra-domain comparisons (Cox regression tests and paired within-subject tests).

**Table 5:** Pairwise inter-domain comparisons results.

Comparison (A vs. B)	TAU RMST	RMST A	RMST B	RMST (B − A)	HR (95% CI)	*p* Cox	*p* PH	*p* Cox FDR	Median TTFI B − A (IQR)	*p* Wilcoxon	*p* Wilcoxon FDR
SCIM vs. UEMS	99	51.512	39.524	−11.988	1.549 (0.742–3.234)	0.244	0.123	0.318	0 (−42–14)	0.288	0.346
SCIM vs. GRASSP Strength	101	51.994	42.429	−9.565	1.388 (0.741–2.600)	0.305	0.055	0.361	0 (−28–14)	0.380	0.415
SCIM vs. GRASSP Sensation	282	95.628	172.365	76.737	0.318 (0.131–0.771)	0.011	0.438	0.029	28 (0–78)	0.010	0.029
UEMS vs. GRASSP-PA	99	39.524	69.600	30.076	0.345 (0.168–0.706)	0.004	0.388	0.016	28 (0–68)	0.012	0.029
UEMS vs. GRASSP-PP	99	39.524	53.667	14.143	0.606 (0.337–1.088)	0.093	0.873	0.173	14 (−12–28)	0.088	0.151
GRASSP Strength vs. GRASSP-PA	101	42.429	70.4	27.971	0.390 (0.210–0.750)	0.004	0.334	0.017	28 (14–56)	0.013	0.03
GRASSP Strength vs. GRASSP-PP	101	42.429	54.157	11.728	0.660 (0.350–1.230)	0.188	0.769	0.257	13 (−13–28)	0.245	0.312
GRASSP Sensation vs. GRASSP-PA	130	100.953	82.000	−18.953	2.133 (0.836–5.443)	0.113	0.593	0.184	0 (−97–14)	0.197	0.296
GRASSP Sensation vs. GRASSP-PP	210	138.538	80.850	−57.688	3.289 (1.345–8.046)	0.009	0.969	0.029	1 (−56–14)	0.007	0.029

Comparisons are based on time to first achievement of the Minimal Clinically Important Difference (MCID). TAU RMST indicates the dynamically defined restricted mean survival time horizon. RMST A and RMST B represent the restricted mean survival time (days) to MCID for measures A and B, respectively; RMST (B − A) denotes the difference between groups (positive values indicate later improvement for B). HR (95% CI) is the hazard ratio from Cox proportional hazards models (HR < 1 indicates earlier MCID achievement for A; HR > 1 indicates earlier achievement for B). *p* Cox refers to the Wald test for the Cox model; *p* PH assesses the proportional hazards assumption; *p* Cox FDR denotes false discovery rate–adjusted *p* values across all pairwise survival comparisons. Median TTFI B − A (IQR) reports the paired within-subject median difference in days; *p* Wilcoxon and *p* Wilcoxon FDR correspond to the signed-rank test and its FDR-adjusted *p* value. Abbreviations: SCIM, Spinal Cord Independence Measure; UEMS, Upper Extremity Motor Score; GRASSP, Graded Redefined Assessment of Strength, Sensation and Prehension; PA, Prehension Ability; PP, Prehension Performance; RMST, Restricted Mean Survival Time; HR, Hazard Ratio; CI, Confidence Interval; FDR, False Discovery Rate. False discovery rate (FDR) correction using the Benjamini–Hochberg procedure was applied across the pairwise inter-scale survival comparisons and paired within-subject comparisons; the columns ‘*p* Cox FDR’ and ‘*p* Wilcoxon FDR’ report the adjusted *p*-values.

**Table 6:** AIS-stratified primary analysis results.

Comparison: Neurologic vs. Functional	TAU RMST	RMST A	RMST B	RMST (B − A)	HR	*p* Cox	*p* PH
AIS C	97	45.900	29.500	−16.400	1.365 (0.657–2.836)	0.405	0.759
AIS D	97	28.273	34.091	5.818	0.869 (0.354–2.136)	0.760	0.470

Restricted mean survival time (RMST)–based and Cox regression results for the comparison between neurologic and functional domains, stratified by American Spinal Injury Association Impairment Scale (AIS) grade (AIS C and AIS D). TAU RMST indicates the dynamically defined truncation time for RMST estimation. RMST A and RMST B represent the mean time to first clinically meaningful improvement (time to first improvement, TTFI) within τ for the neurologic and functional domains, respectively; RMST (B − A) reflects the average delay or anticipation of functional relative to neurologic improvement (negative values indicate earlier neurologic recovery). HR denotes the hazard ratio from Cox proportional hazards models (functional domain as reference), with values >1 indicating a higher instantaneous probability of reaching minimal clinically important difference (MCID) in the neurologic domain. *p* Cox refers to the Wald test for the hazard ratio, and *p* PH reports the test for proportional hazards assumption.

**Table 7:** Dominant vs. Non-dominant limb analysis results.

Comparison: Dominant vs. Non-Dominant	TAU RMST	RMST A	RMST B	RMST Diff B Minus A	HR	*p* Cox	*p* PH
UEMS	99	48.857	52.798	3.940	0.886 (0.534–1.470)	0.640	0.559
GRASSP Strength	101	51.238	46.429	−4.810	1.209 (0.944–1.548)	0.132	0.746
GRASSP Sensation	282	192.125	187.285	−4.840	1.184 (0.575–2.435)	0.647	0.892
GRASSP-PA	130	92.095	94.520	2.425	0.914 (0.513–1.629)	0.761	0.395
GRASSP-PP	210	99.418	91.720	−7.698	1.027 (0.650–1.624)	0.908	0.445

Kaplan–Meier and Cox regression analyses comparing time to first clinically meaningful improvement (TTFI) between dominant and non-dominant upper limbs for limb-specific outcome measures. TAU RMST indicates the restricted mean survival time horizon; RMST A and RMST B represent the restricted mean time (days) to minimal clinically important difference (MCID) for the non-dominant (A) and dominant (B) limb, respectively; RMST diff (B − A) reflects earlier (negative) or later (positive) improvement in the dominant limb. HR denotes the hazard ratio for MCID achievement (dominant vs. non-dominant), with values >1 indicating faster improvement in the dominant limb. *p* Cox refers to the Cox proportional hazards test; *p* PH assesses the proportional hazards assumption. No comparisons showed statistically significant differences, indicating broadly symmetrical recovery trajectories between limbs. Acronyms: UEMS = Upper Extremity Motor Score; GRASSP = Graded Redefined Assessment of Strength, Sensation and Prehension; GRASSP-PA = GRASSP Ability; GRASSP-PP = GRASSP Performance; RMST = Restricted Mean Survival Time; HR = Hazard Ratio.

## Data Availability

Data available on request.

## Abbreviations

ABR	Activity-Based Rehabilitation
ADLs	Activities of daily living
AIS	American Spinal Injury Association Impairment Scale
Cis	Confidence Intervals
FDR	False Discovery Rate
FLS	I.R.C.C.S. Fondazione Santa Lucia
GDPR	General Data Protection Regulation
GRASSP	Graded Redefined Assessment of Strength, Sensation and Prehension
GRASSP-PA	Prehension Ability
GRASSP-PP	Prehension Performance
HRs	Hazard ratios
ISNCSCI	International Standards for Neurological Classification of Spinal Cord Injury
IQR	Interquartile Range
MCD	Minimal Detectable Change
MCID	Minimal Clinically Important Difference
MID	Minimal Important Difference
RMST	restricted mean survival time
SCI	Spinal Cord Injury
SCIM	Spinal Cord Independence Measure II
TAU RMST	Truncation Time Used for Restricted Mean
TTFI	time to first clinically meaningful improvement
UEMS	Upper Extremity Motor Score
